# Association of the Microbiota and Pancreatic Cancer: Opportunities and Limitations

**DOI:** 10.3389/fimmu.2022.844401

**Published:** 2022-03-03

**Authors:** Zhou Chen, Shaofeng Zhang, Shi Dong, Hao Xu, Wence Zhou

**Affiliations:** ^1^ Department of General Surgery, The First Hospital of Lanzhou University, The First Clinical Medical School of Lanzhou University, Lanzhou University, Lanzhou, China; ^2^ The First Clinical Medical College, Lanzhou University, Lanzhou, China; ^3^ Department of General Surgery, Gansu Provincial Hospital, Lanzhou, China

**Keywords:** microbiota, biomarkers, molecular mechanism, targeted therapy, pancreatic cancer

## Abstract

The human body is thoroughly colonized by a wide variety of microorganisms, termed microbiota. Pancreatic cancer, one of the most aggressive forms of cancer, is no exception. The microbiota of pancreatic cancer largely influences and even dominates the occurrence, development and outcome of pancreatic cancer in many ways. Studies have shown that microbiota could change the malignant phenotype and prognosis of pancreatic cancer by stimulating persistent inflammation, regulating the antitumor immune system, changing the tumor microenvironment and affecting cellular metabolism. This is why the association of the microbiota with pancreatic cancer is an emerging area of research that warrants further exploration. Herein, we investigated the potential microbial markers of pancreatic cancer, related research models, the mechanism of action of microbiota in pancreatic cancer, and pancreatic cancer-microbiota-related treatment.

## Introduction

Pancreatic cancer (PC) is one of the most malignant tumors of the digestive tract and the third most deadly cancer globally (1). The onset of PC is insidious, and most patients are already at an advanced stage at the time of diagnosis, often accompanied by distant metastases. In recent years, the survival time of patients with PC has improved to a certain extent. Nevertheless, the outcome remains unsatisfactory, with the 5-year survival rate of patients being lower than 10% (1). At present, radical surrgery is still the mainstay treatment strategy for PC. Unfortunately, less than 20% of PC patients are suitable for resection (2). In addition, patients with PC have a relatively high recurrence rate after surgery, and most patients will eventually succumb due to metastasis (3). The available treatment options for other advanced PC patients are very limited. However, if the primary tumor is less than 2cm in diameter and is confined to the pancreas upon early detection, the 5-year survival rate of patients can reach up to 46% after surgical treatment (4, 5). Therefore, the early detection of PC lesions and the improvement of late treatment are very critical. It is generally believed that changes in the genome of various cancers can effectively and accurately predict patient survival time and response to chemotherapy drugs (6). However, among PC patients with the same tumor grade, investigators have found no significant genomic differences between patients with long- and short term overall survival (7). To date, the factors contributing to the different survival outcomes are still complex and elusive. Innovative approaches for early screening, prevention, and treatment of PC are thus urgently needed.

Any part of the human body connected to the outside world can become a custom home for millions of microbiota, with the digestive tract being the preferred site for colonization (8), housing roughly 10^13^~10^14^ gut microbiota, including bacteria, archaea, fungi, protists, and viruses, with bacteria being the primary inhabitants, consisting of at least 100 times as many genes as the human genome (8). Under physiological conditions, many colonies maintain a dynamic balance of mutual benefits with the human body. For example, the human body provides the nutrients required by the microbiota and a suitable living environment, and in return, the microbiota synthesizes and provides the essential amino acids and vitamins that our bodies need as well as processes other indigestible components of our diet, such as plant polysaccharides (9). As a new emerging research field, the human microbiota plays an important role in various gastrointestinal cancers, such as esophageal cancer, with reports suggesting that 16% of malignant diseases are associated with microbial infections; and this percentage is even higher in digestive tract-related malignancies such as liver and gastric cancers, being as high as 80% (1). In these malignancies, the gut microbiota is in direct contact with high-risk organs. This article reviews the potential microbial markers of PC, related research models, the mechanism of action of microbiota in PC, and pancreatic cancer-microbiota-related treatment.

## Microbiota Colonization Pathways in Pancreas

**Figure 1:**
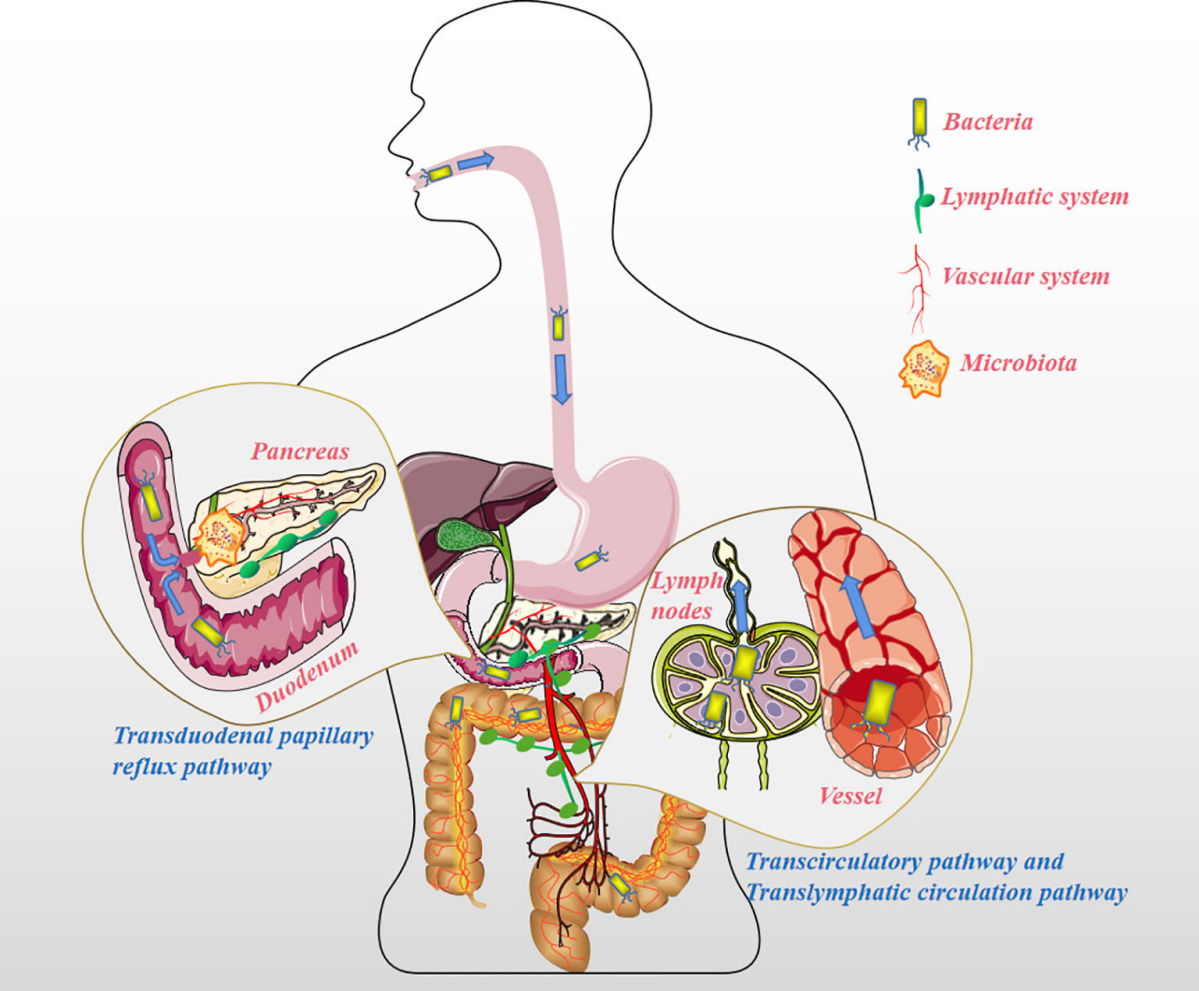
The pathways the microbiota migrates to the pancreatic tissue. Transduodenal papillary reflux pathway: The pancreas is connected to the oral cavity, esophagus, and stomach upward through the pancreatic duct, downward to the duodenum, and adjacent to the common bile duct. These features suggest the possibility of microbiota reflux into the pancreatic duct and then into the pancreatic parenchyma through the large/little papillae. Translymphatic circulation pathway: The microbiota distant from the pancreas (such as in the colon) enters the MLN by chance and is phagocytized by mononuclear phagocytes (e.g. CX3CR1^+^ cells) or DCs, and instead of being lysed, these opportunistic pathogens are fortunately transferred to the pancreatic tissue for reproduction *via* the lymphatic system. Transcirculatory pathway: The microbiota far away from the pancreas (such as in the colon) enters the blood under pathological conditions (such as damage to the intestinal barrier caused by colitis), and colonizes other organs, including pancreatic tissue, along with the blood circulation.

The pancreas, previously considered sterile, has been increasingly shown to be colonized by several microorganisms, a finding that is independent to the state of the pancreas (normal or pathological) (10, 11). For instance, Geller et al. detected bacteria in 113 pancreatic ductal adenocarcinoma (PDAC) samples using the 16S ribosomal RNA (16S rRNA) gene sequencing method and found that 86 (76%) of them were positive for bacterial presence, among the 20 normal pancreas controls, only 3 (15%) contained bacterial DNA (P <0.005) (12). The presence of microbiota in PC has been recognized and accepted by many scholars, but it remains unclear how bacteria colonize the pancreas. Throughout the history of research on PC and microbiota, the pathways by which microbiota colonize PC tissue can be summarized as follows (**Figure 1**). Firstly, the pancreas is connected to the oral cavity, esophagus, and stomach upwards through the pancreatic duct, downwards to the duodenum, and adjacent to the common bile duct. These features confer the possibility of bacterial reflux through the pancreatic duct and eventually into the pancreatic parenchyma through the large/little papilla (13). In a study by Pushalkar et al., wild-type mice were administered CFSE-labeled *E. faecalis* (2.5×10^8^ CFU) *via* oral gavage, after which the mouse pancreatic tissue was extracted after 0.5h, and the presence of CFSE-labeled *E. faecalis* was detected. The same results were observed for GFP-labeled *E. coli* (2.5×10^8^ CFU) administered *via* oral gavage in mouse pancreatic tissue (14). Similarly, Aykut et al. administered GFP-labeled *Saccharomyces cerevisiae* to control or tumor-bearing mice *via* oral gavage, and fungi were detected in pancreatic tissue within 30 minutes (15). The author hypothesized that it might be due to the reflux phenomenon allowing bacteria to enter *via* the pancreatic duct (16). However, in another mouse model, GF 129SvEv mice were orally gavaged with *Campylobacter jejuni*, and pancreatic tissue was collected after 1, 2, 4, and 8 weeks of housing, and no evidence of pancreatic colonization by the microbiota was detected (10), the reason might be that the acquisition of pancreatic bacteria is not a physiological process, but a pathological process (10). Regardless, the above anatomical features of the pancreatic duct create ideal condition for bacterial colonization of the pancreas. Secondly, it seems reasonable that the microbiota of the upper gastrointestinal tract could enter the pancreas by reflux through the large/little papilla, while microbiota situated further, such as in the colon, may migrate to the pancreas through other pathways. In antibiotic-treated mouse model experiments, Diehl et al. uncovered that non-invasive *Salmonella* was carried into the mesenteric lymph node (MLN) by the CX3CR1^+^ cell (one of the CD11c^+^ mononuclear phagocytes, the other being CD103^+^ cell) (17). To confirm this conclusion, they sorted CX3CR1^+^ and CD103^+^ cells from the MLN of infected antibiotic-treated mice and assayed for cells containing colony-forming units; bacteria were detected only in CX3CR1^+^ cells but not in CD103^+^ cells. In addition, CX3CR1^+^ cells have the ability to migrate from the small intestine to the MLN. However, at a steady-state, the transport of symbiotic bacteria and pathogenic bacteria from the lumen to the MLN is restricted, and MLN is a key immune induction site (18). Using a mouse model infected with *S. enterica* serovar *Typhimurium* (STM), Bravo-Blas et al. detected dendritic cells (DCs) in lymphocytes collected from mouse thoracic ducts, including CX3CR1^int^ and CX3CR1^lo^, which is contrary to the experimental results of Diehl et al. In addition, STM is not only transported by DCs but also autonomously. After reaching the MLN, STM can be taken up by macrophages, DCs and some B cells (19). Whether the migration of intestinal flora is associated with intestinal permeability remains debatable (10). When the digestive tract flora is dysfunctional, opportunistic pathogenic bacteria can invade the MLN to be captured by DCs. DCs containing opportunistic pathogens might become a good vehicle for the systemic dissemination of pathogenic bacteria. This implies that the gut microbiota may colonize pancreatic tissue *via* the lymphatic system pathway. Lastly, in a feline model of acute pancreatitis, Widdison et al. demonstrated that *E. coli* could spread to the pancreas through the bloodstream, the colon wall, and enter the pancreatic duct through reflux (20). Studies also show that a high-fat diet could induce the transfer of bacteria from the intestine to human blood (21). Sato et al. analyzed the fecal and blood flora of 50 patients with type 2 diabetes compared to healthy subjects and discovered the presence of intestinal flora dysbiosis and transfer of bacteria from the intestine to the bloodstream in patients with type 2 diabetes (22). Factors such as a change in eating habits, the use of antibiotics, or the flora’s metabolites could also change the intestinal permeability and/or destroy the intestinal barrier function, creating an opportunistic environment for gut flora to enter the circulation (23–27). In addition, a growing number of research have pointed to the fact that specific microbiota is also detected in healthy human blood (28). Since studies have shown that microbiota is already present in placental tissue and cord blood, microbiota migration to pancreatic tissues may occur at an even earlier stage (29–32).

## Potential Microbial Markers in PC

### Oral Microbiota and PC

**Figure 2:**
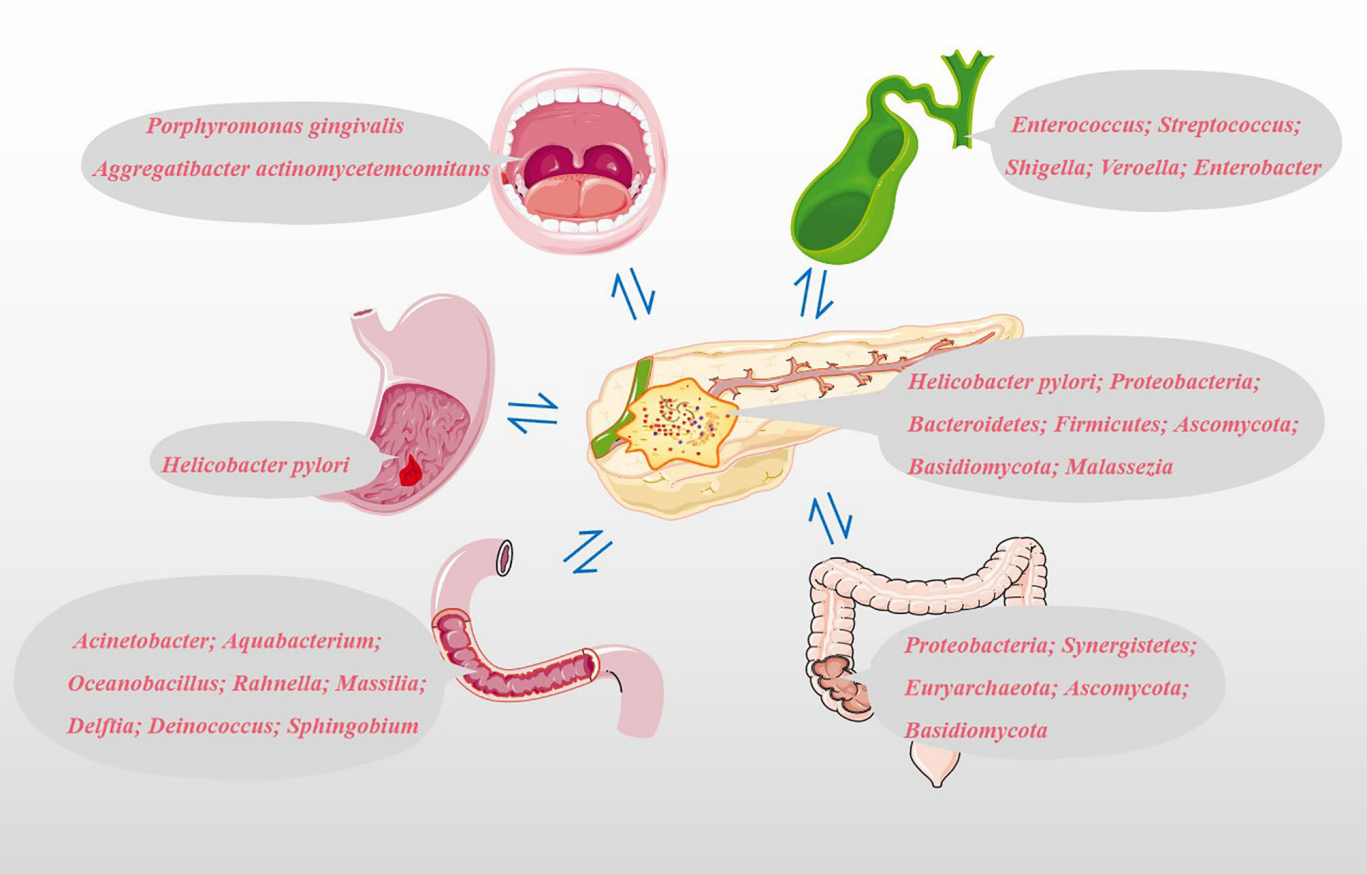
Potential microbial markers associated with PC. Oral microbial markers: *Porphyromonas gingivalis* and *Aggregatibacter actinomycetemcomitans*; Gastric microbial markers: *Helicobacter pylori*; Duodenal microbiota markers: *Acinetobacter*, *Aquabacterium*, *Oceanobacillus*, *Rahnella*, *Massilia*, *Delftia*, *Deinococcus* and *Sphingobium*; Common bile duct microbial markers: *Enterococcus*, *Streptococcus*, *Shigella*, *Veroella* and *Enterobacter*; Colonic microbial markers: *Proteobacteria*, *Synergistetes*, *Euryarchaeota*, *Ascomycota* and *Basidiomycota*; Pancreatic microbial markers: *Helicobacter pylori*, *Proteobacteria*, *Bacteroidetes*, *Firmicutes*, *Ascomycota*, *Basidiomycota* and *Malassezia*.

Based on the possible pathways that we have mentioned above, the microbiota from different sites could colonize the pancreatic tissue and directly or indirectly play a role in the development and progression of PC. Specimens obtained from different parts of our bodies, such as oral saliva, serum, feces and pancreatic tumor tissues, are shown in **Figure 2**. More than 700 different species of bacteria colonize the human mouth (33), playing an important role in the immune response, metabolism of carcinogens and digestion of nutrients (34, 35). Michaud et al. measured antibodies against 25 oral bacteria in pre-diagnostic blood samples from 405 PC cases and 416 matched controls, with the results revealing that individuals with high levels of antibodies against *Porphyromonas gingivalis* ATTC 53978 were twice as likely to develop PC compared to individuals with lower levels of these antibodies (OR, 2.14; 95% CI, 1.05-4.36) (36). In a 10-year prospective randomized controlled study, Fan et al. utilized 16S rRNA gene sequencing to comprehensively screen oral microbiome in 361 PC cases and 371 matched control oral wash samples (10 ml scope mouthwash). They demonstrated that the presence of *P. gingivalis* and *A. actinomycetemcomitans*, and decreased relative abundance of phylum *Fusobacteria* and its genus *Leptotrichia* were related to subsequent increased risk of PC (37). The result of a meta-analysis shown that high levels of antibodies to *Porphyromonas gingivalis* are associated with a three-fold increased risk of digestive cancers, including PC (38). Subsequent studies have shown that the intracapsular pancreatic microbiome contains symbiotic bacteria known to inhabit the human oral cavity (39). It is still unclear whether the observed microbiome features precede and contribute to carcinogenesis or whether they develop following cancer development. In addition, other PC etiological factors such as smoking and alcohol consumption could affect the structure of the oral microbial community (37). For example, more dangerous pathogenic bacteria *P. gingivalis* and *A. actinomycetemcomitans* reside in the mouths of PC patients who smoke or drink alcohol (37).

### Gut Microbiota and PC

The relationship between *Helicobacter pylori* and PC is still controversial. Risch et al. provided evidence for the association between *Helicobacter pylori* seropositivity and increased PC risk using enzyme-linked immunosorbent assay (ELISA). Besides, this study also confirmed the association between PC and non-O blood types and shown that the impact of *Helicobacter pylori* seropositivity is particularly pronounced in non-O individuals. This was even more pronounced in individuals who were CagA-negative and *H pylori* seropositive (OR: 2.78, 95% CI: 1.49 -5.20, P =0.0014) (40). However, some reports also found no association between *Helicobacter pylori* infection and PC risk in western European populations (41). Discussion of the association between *Helicobacter pylori* and PC using serum *Helicobacter pylori* DNA levels seems less convincing than direct detection of *Helicobacter pylori* in PC tissue. Using a Helicobacter-specific PCR assay, *Helicobacter pylori* was previously detected in PC tissues and/or adjacent tissues in 75% of patients with exocrine PC, while all samples from other benign pancreatic diseases and normal pancreas were negative (42). Presently, the increased risk of PC due to *Helicobacter pylori* is more widely accepted by scholars.

Pushalkar et al. analyzed bacterial membership and structure in stool samples from pancreatic ductal adenocarcinoma (PDA; n=32) versus matched healthy individuals (n=31). At the phylum level, *Firmicutes* and *Bacteroidetes* were predominant in the feces of PDA patients and healthy controls. However, *Proteobacteria*, *Synergistetes*, and *Euryarchaeota* were significantly more abundant in PDA patients compared with healthy subjects. 16S rRNA gene sequencing of PDA tumors from 12 of 32 patients with PDA shown that *Proteobacteria* (45%), *Bacteroidetes* (31%) and *Firmicutes* (22%) were the most prevalent in all samples (14). By amplifying the ITS1 region of the 18S rRNA gene, Aykut et al. tested the feces and tumor fungal communities of PDA patients and found that *Ascomycota* and *Basidiomycota* were the most common phyla in the intestine and tumor tissues, and compared with the intestine, *Malassezia* was more common in tumor tissues (15). The five most important genera in bile are *Enterococcus*, *Streptococcus*, *Shigella*, *Veroella*, and *Enterobacter* (43). *Acinetobacter*, *Aquabacterium*, *Oceanobacillus*, *Rahnella*, *Massilia*, *Delftia*, *Deinococcus*, and *Sphingobium* are more abundant in the duodenal mucosa of patients with PC (43). The above microbiota are closely related to the occurrence and development of PC and may be used as biomarkers for the non-invasive diagnosis of PC.

### Microbiota in Pancreatic Tumor

Using an aseptic technique, Thomas et al. obtained normal pancreatic tissue (n=7), pancreatitis tissue (n=4) or PDAC (n=16). They found that microbiota was found in all, but no differences in flora were detected in these tissue samples. In subsequent *in vivo* experimental colony transplantation experiment, the author failed to find evidence of pancreatic colony transplantation of the orally administered microbiota (*Campylobacter jejuni*) (10). The microbiota is not only different in benign and malignant tumors but also dependent on the use of neoadjuvant treatment or not, whether the tumor has recurred, the length of patient survival, and ratio of tumor parenchyma to mesenchyme (44–48). Riquelme et al. tried to assess the difference in the average relative abundance of microbial species between long-term survivors (LTS) and short-term survivors (STS), found that *Pseudoxanthomonas*, *Streptomyces*, *Saccharopolyspora* and *Bacillus clausii* were significantly enriched in LTS cohort, which may contribute to predicting survival time after PC surgery and be potential to be prognostic biomarkers (48). Studies have shown that the microbiota might induce the development of PC. Intraductal papillary mucinous neoplasm (IPMN) is most common among pancreatic cystic neoplasms (PCN) (49) and could develop into invasive carcinoma (50). Compared with low-grade dysplasia IPMN, high-grade dysplasia IPMN tissue is colonized with more bacteria and is more diverse with sample dominated by either *Firmicutes* or *Proteobacteria* at the phylum level (39). However, a proportion of PC patients with preoperative biliary obstruction require ERCP, implantation of a ballistic stent and use of antibiotics, which could cause artificial reflux infections and inevitable pancreatic tissue microbiota transplantation in the short term, affecting the results of the microbiota identification in collected specimens (39, 47). In addition, only some pathogenic bacteria can be traced due to the large variety of microbiota and temporal and spatial variations. Microbiota on PC is summaried in **Table 1**.

**Table 1:** Studies on the association between microbiota and pancreatic cancer.

Study author, year of publication	Sample size (Tumor group vs non-tumor group)	Microbiome specimen	Microbial alterations (increases)	Microbial alterations (decreases)	Significance
Fan et al., 2018 (37)	361, 371	Oral wash samples	*P. Gingivalis*, *A. actinomycetemcomitans*	*Fusobacteria*, *Leptotrichia*	Increased risk of PC
Risch et al., 2010 (40)	373, 690	Serum	*H pylori*		Increased risk of PC
Pushalkar et al., 2018 (14)	32, 31	Fecal and PDA	*Proteobacteria*, *Synergistetes*, *Euryarchaeota*, *Bacteroidetes*, *Firmicutes*		Increased risk of PC
Aykut et al., 2019 (15)	13, 5	Fecal and PDA	*Ascomycota*, *Basidiomycota*, *Malassezia*		Increased risk of PC and promoted tumor progression
Mei et al., 2018 (43)	14, 14	Bile	*Enterococcus*, *Streptococcus*, *Shigella*, *Veroella*, *Enterobacter*		Increased risk of PC
Mei et al., 2018 (43)	14, 14	Duodenal mucosa	*Acinetobacter*, *Aquabacterium*, *Oceanobacillus*, *Rahnella*, *Massilia*, *Delftia*, *Deinococcus*, *Sphingobium*		Increased risk of PC
Gaiser et al., 2019 (39)	14, 8	IPMN	*Firmicutes*, *Proteobacteria*		Increased risk of PC and promoted tumor progression
Geller et al., 2017 (12)	113, 20	PDAC	*Enterobacteriaceae*, *Pseudomonadaceae*		Increased drug resistance
Riquelme et al., 2019 (48)	22, 21	PDAC	*Pseudoxanthomonas*, *Streptomyces*, *Saccharopolyspora*, *Bacillus clausii*		Predicted long-term survivor

## Potential Pathogenic Mechanisms of Microbiota in PC

### Cytotoxicity and Pro-Inflammatory Effects of Microbiota

**Figure 3:**
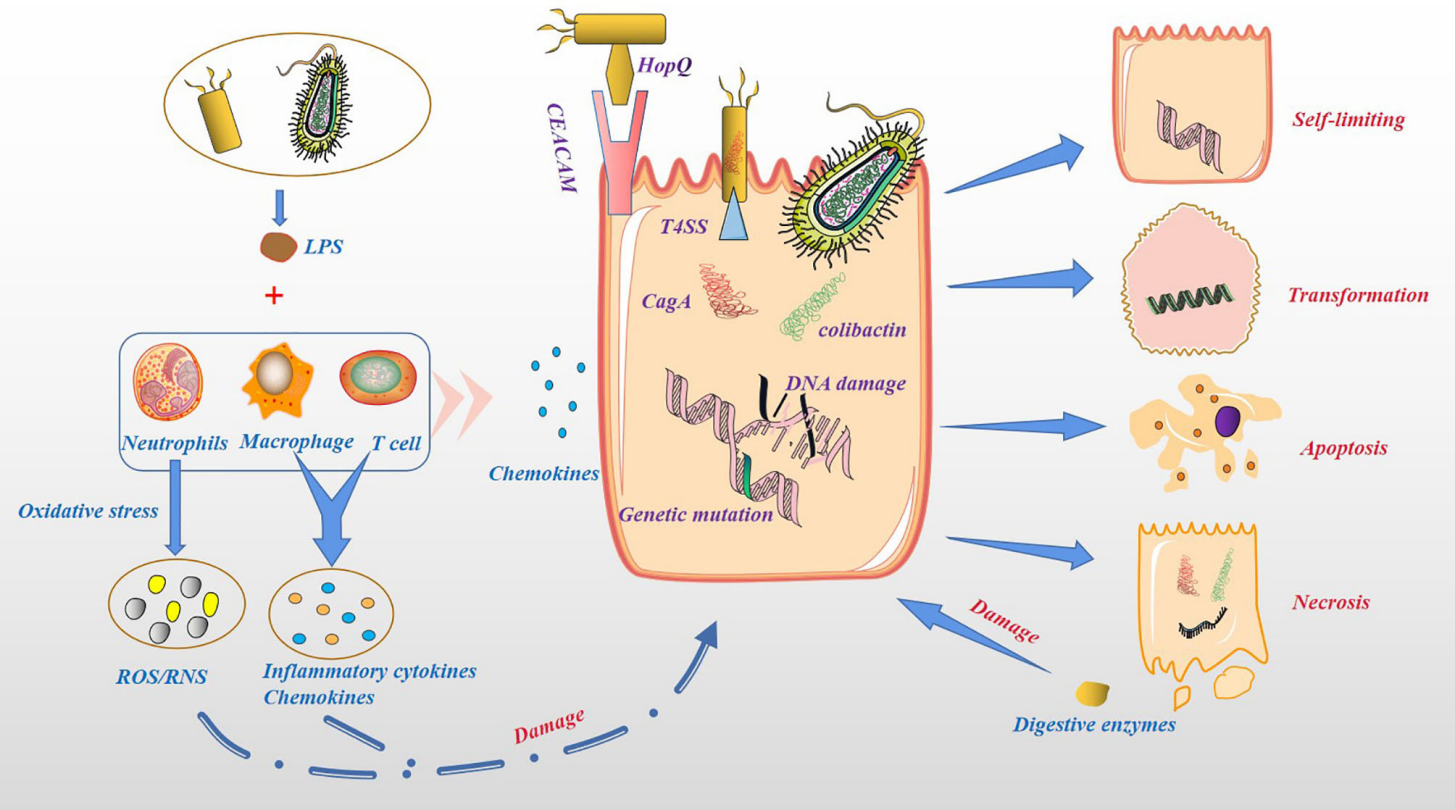
Cytotoxicity and pro-inflammatory effects of microbiota. Bacteria can act directly on host cells and produce toxic effects. For example, *Helicobacter pylori* binds to gastric epithelial cells through the adhesin HopQ and CEACAM, and the virulence factor CagA is directly injected into epithelial cells through T4SS. Colibactin secreted by Pathogenic *Escherichia coli* in the host cellinduces cross-links between DNA strands and double-strand DNA breaks. Host cells secrete chemokines and recruit immune cells, such as neutrophils, macrophage and T cells. These immune cells are activated by LPS to produce ROS/RNS, inflammatory cytokines and chemokines, increase damage to host cells. Damaged host cells may be transformed, self-limiting, apoptosis or necrosis, while necrotic pancreatic cells release endogenous digestive enzymes, further damaging other pancreatic cells.

Based on the above, bacterial colonization of the pancreatic tissue can occur through multiple pathways. The acceptance of the microbiota as a foreign body by the pancreatic tissue depends on the biological effects produced by the microbiota (**Figure 3**). As we all know, the proliferation of bacteria is much faster than the proliferation of human cells, producing more metabolites, some of which are beneficial to the human body, while others are harmful. The nutrient supply in pancreatic tissue is not like in the intestines, which provides most of the nutrients through oral food. In the case of food insufficiency, the microbiota may prey on pancreatic tissue cells to obtain nutrients. For example, *Helicobacter pylori* can bind to gastric epithelial cells through the adhesin HopQ and carcinoembryonic antigen-related cell adhesion molecules (CEACAM) and the virulence factor CagA is directly injected into epithelial cells through type 4 secretion system (T4SS). CagA could ultimately activate the Wnt/β-catenin pathway, leading to cell turnover and apoptosis (51). Pathogenic *Escherichia coli* has a complete set of virulence factors and toxins related to pathogenicity, including secretory genetic poison colibactin. Once in the host cell, colibactin induces cross-links between DNA strands and double-strand DNA breaks (51). Pancreatic tissue could counter this malignant effect induced by the microbiota through an inflammatory response. In addition, the injured pancreatic alveolar cells can release endogenous digestive enzymes that further aggravate cellular damage (52, 53). In human and animal models, the dysbiosis of the gut microbiota is related to the severity of acute pancreatitis (AP) (11). This seems to depend on the activation of NOD-like receptor protein 3 (NLRP3), an intracellular pattern recognition molecule that detects microbial and hazard-related molecular patterns (54, 55). Moreover, the recruitment of neutrophils (56), macrophages (57) and pro-inflammatory mediators such as IL-6 (58) also play a key role. Although most AP episodes are mild and self-limiting, some can progress to chronic pancreatitis (59), especially those with recurrent AP. In the past two decades, PC has been considered to be an inflammation-driven cancer, and patients with chronic pancreatitis have a higher risk of PC (60, 61). Chronic pancreatitis and its mechanism in causing PC are still unclear. At present, the research on microbiota and chronic pancreatitis in immunity has made great progress. For instance, the microbial component lipopolysaccharide (LPS) can effectively activate the host’s innate immune system (62). In chronic pancreatitis, T cells and macrophages are the main immune infiltrating cells (63, 64), thereby impairing the regeneration of pancreatic cells and promoting the dedifferentiation of the pancreatic epithelium (57, 65), and conferring the potential for pancreatic epithelial cells to progress to cancer cells (66). The reason for this is that microbiota can induce a sustained inflammatory response, in which oxidative stress and the generation of reactive oxygen species (ROS), reactive nitrogen species (RNS), immune cells and other stromal components, such as endothelial cells and pancreatic stellate cell (PSC) play key roles (67). On the one hand, ROS/RNS lead to DNA fragmentation, membrane disassembly and protein misfolding through modification of key substrates, such as nucleic acids, lipids and preproteins. On the other hand, inflammatory cytokines and chemokines produced by immune cells and other stromal components work together with ROS/RNS to aggravate epithelial cell damage and increase proliferation (67). In addition, Inflammatory mediators, such as Cyclooxygenase-2 (Cox2), NF-κB and STAT3, might promote the development of chronic inflammation and preneoplastic lesions (68, 69). However, the above experiments in animal models of chronic pancreatitis transforming into PC result from a combination of activation of oncogenic Kras and loss of the tumor suppressor barrier and tissue damage produced by the inflammatory response. In other words, the most fundamental cause of PC is the change in the expression level of cellular oncogenes. Furthermore, the microbiota can cause DNA fragmentation and protein misfolding, resulting in an increase in the level of oncogenes and the malignant phenotype of tumor cells.

### Microbiota Metabolites in PC

**Figure 4:**
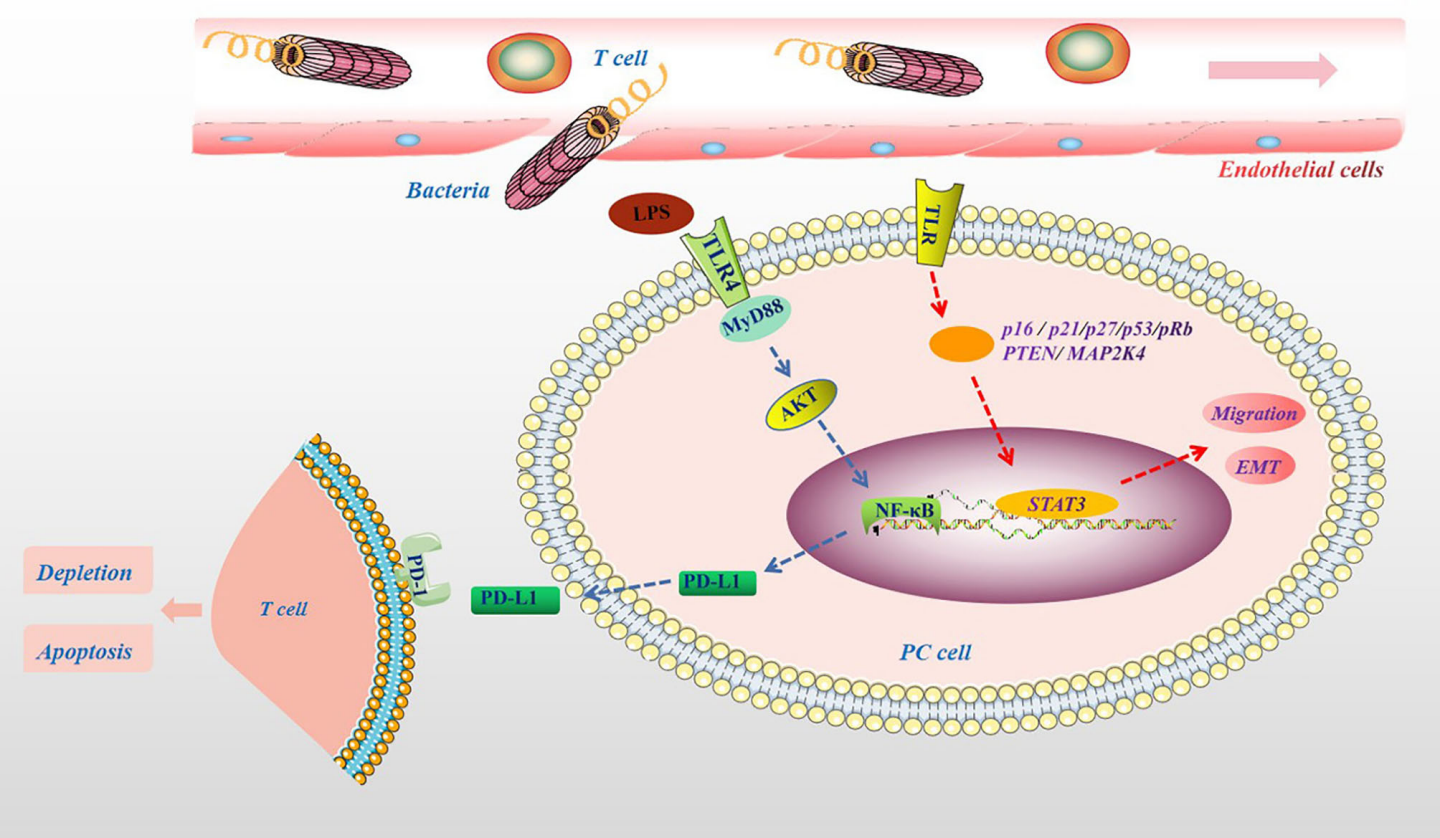
The pathogenic molecular mechanisms of microbial metabolites in PC. Microbial lysates such as LPS in the pancreatic TME, upregulates PD-L1 through the TLR4/MyD88/AKT/NF-κB signaling pathway, and induces TILs depletion and apoptosis. The activation of TLRs can cause a variety of tumor suppressor proteins, such as p16, p21, p27, p53, pRb, PTEN and MAP2K4, disorder, which induce STAT3 activation, promote migration and EMT.

The important influence of the microbiota on the human body is mainly reflected in the metabolism of ingested sugar, fat, and protein and the synthesis of vitamins and other nutrients (70). In addition, the microbiota and their metabolites participate in physiological and pathological processes in the body, including cell proliferation, differentiation, apoptosis, tumor development and aggressiveness (71, 72). For example, the microbiota can cause changes in the body’s metabolism, leading to various metabolic diseases such as obesity and diabetes. Obesity and diabetes are also important risk factors for the development of PC (73). In particular, a high-fat and high-energy diet promotes the absorption of harmful metabolites of the microbiota, such as bacterial LPS, into the circulation (74). This may be because the microbiota can affect the metabolism of carbohydrates and the production of short-chain fatty acids (SCFAs), damaging the tight junctions of the intestinal mucosal epithelium and promoting bacterial endotoxins to enter the bloodstream (75). Clinical results also show that there were more LPS-producing bacteria in the intestine of PC patients (76). In addition, in PC tissues, the main bacterial groups detected, such as *Proteobacteria* and *Bacteroidete*, belong to gram-negative bacteria containing LPS (14). More importantly, many LPS-containing bacteria exist in the microenvironment of PC tumors (12). It is currently believed that LPS is a specific agonist that triggers the Toll-like receptor 4 (TLR4) signaling pathway in immune cells (77). Studies have shown that TLR4 is also highly expressed in various cancer cells, including PC cells, and may promote the proliferation and invasion of PC through the up-regulation of HIF-1α and is closely related to prognosis (78). Recent experimental results show that the destruction of the intestinal barrier induces high circulating LPS and increases LPS deposition in tumor tissues. In the early stage, LPS can significantly infiltrate CD3^+^ and CD8^+^ T cells, inhibiting tumor growth, while long-term induction leads to depletion of T cells. Additionally, LPS upregulates programmed cell death ligand 1 (PD-L1) through the TLR4/MyD88/AKT/NF-κB signaling pathway and induces the depletion and apoptosis of tumor-infiltrating lymphocytes (TILs), thereby promoting cancer immune evasion (79). In addition, the activation of TLRs can inactivate a variety of tumor suppressor proteins (such as p16, p21, p27, p53, pRb, PTEN and MAP2K4), inducing STAT3 activation, promoting epithelial-mesenchymal transition (EMT), PC cell migration and oncogene-induced senescence (80, 81), which are shown in **Figure 4**.

### Microbiota and Tumor Micro-Immune System

**Figure 5:**
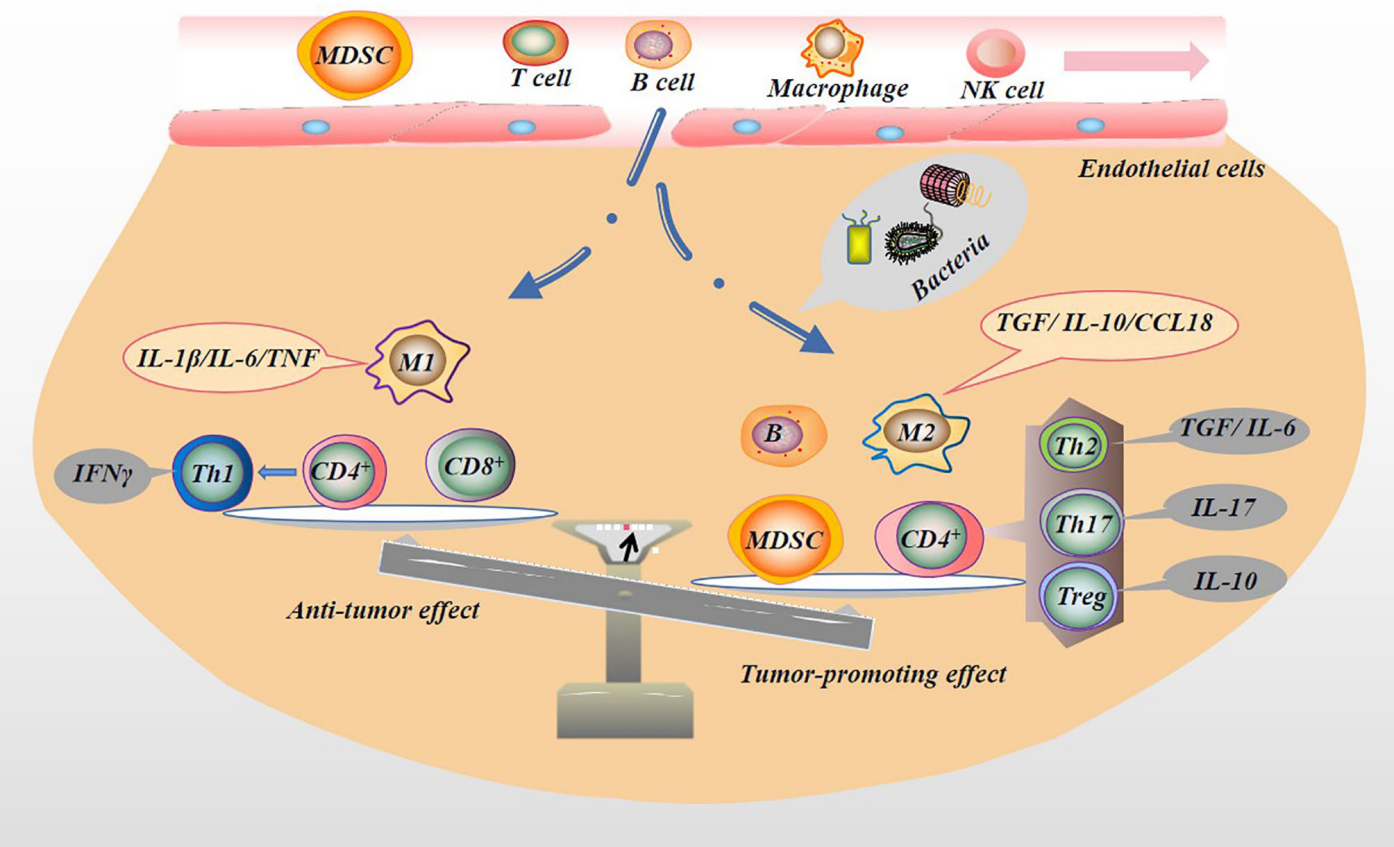
Microbiota regulates tumor immune microenvironment. The microbiota in the TME can activate the immune system and recruit immune cells. Immune cells are induced by microbiota to differentiate into different subtypes of immune cells, which secrete the appropriate factors that play pro- or anti-neoplastic roles in tumorigenesis and progression. For example, immune cells with antitumor effects, M1 macrophages (secreting IL-1β/IL-6/TNF), CD8^+^ T cells, and Th1 (differentiated from CD4^+^ T cells and secreting IFNγ) may be reduced by the presence of microbiota. In contrast, immune cells with pro-tumor effects, M2 macrophages (secreting TGF/IL-10/CCL18), B cells, MDSC, and CD4^+^ differentiated into Th2 (secreting TGF/IL-6), Th17 (secreting IL-17), and Treg (secreting IL-10) are increased.

Immune cells, including T cells, natural killer (NK) cells, macrophages, and DCs, play an important role in inhibiting tumor initiation and progression. NK cells are a type of effector cells that act in the early stage of tumor and are the body’s first line of defense against tumors. But NK cells are mostly located in circulation. In solid tumor tissues, infiltrating NK cells are rarely seen (82). NK cells monitor tumor cells in the blood circulation and prevent the metastasis of tumor cells. When NK cells are depleted or inhibited, tumor growth and escape might result (83, 84). The entry of the microbiota into the circulation and the release of inflammatory factors may cause the consequences mentioned above, especially in patients with advanced PC or cachexia. It remains debatable whether NK cells can facilitate solid tumor infiltration because they inhibit CD8^+^ T cell responses during chronic infections (85). Indeed, activation of NK cells enhances immune pathology and promotes chronic infection by limiting CD8^+^ T cell immunity (85). T cell response is the most important host response that controls tumor growth and development, and it is also an important immune cell. In non-lymphatic tissues, the tumor endothelial barrier composed of resting endothelial cells in capillaries and venules makes it hard for immune cells to enter the tissue. When an infection occurs, endothelial cells, parenchymal cells and epithelial cells produce chemokines, which together with proteolytic fragments attract a subpopulation of immune cells. For example, once a few T cells infiltrate the tumor and express chemokines, they attract a large influx of specific and non-specific T cells (86). CD4^+^ and CD8^+^ T cells play different roles in the tumor microenvironment (TME). For example, helper T cell (Th cell), including the Th2, Treg or Th17 lineages, differentiated by CD4^+^ T cells, play a tumor-promoting role in tumors (87, 88). After activation, CD8^+^ T cells differentiate into CTLs, which have antitumor effects. Mouse model experiments proved that PC mice enriched with CD8^+^ T cells survived longer (89). In PC patients, tissues infiltrated by CD8^+^ T cells shown a longer survival time (90). However, the microbiota dominates and regulates the ratio of CD4^+^ to CD8^+^ T cells in the PC TME. In mouse model experiments, Pushalkar et al. proved that the ratio of CD8^+^: CD4^+^ T cells in PC tissues increased after microbial ablation. Not only that, but microbial ablation also enhanced the Th1 polarization and cytotoxicity of CD4^+^ T cells and CD8^+^ T cells phenotype acquisition (14). In the more malignant basal-like tumor tissue, more memory B cells were found. This result may be attributed to the pathogen’s excessive immune response and inflammation (91). B cells play a role in supporting the growth of PC cells (92). Myeloid-derived suppressor cells (MDSCs) are immature bone marrow cells, a heterogeneous population of immature bone marrow cells containing common precursors from DCs, macrophages, and granulocytes, which are increased in the circulation, bone marrow, and spleen of tumor-bearing mice and tumor patients, and contribute to tumor cells escaping the antitumor immune response (93). MDSCs reduce the proliferation of CD8^+^ T cells and increase apoptosis by generating ROS and RNS. The consumption of CD8^+^ T cells in mice eliminates the protective effect of complement deficiency on tumor growth (94, 95). Microbial ablation can reduce the proportion of MDSCs in the tumor in the orthotopic KPC model, thereby reducing tumor cell immune evasion (14). Microbial ablation resulted in a decrease in immunosuppressive CD206^+^ M2-like TAM with a concomitant increase in M1-like tumor-associated macrophages (TAMs), while at the same time M1-like TAM increased, with higher expression of MHC II, CD86, TNF-α, IL-12 and IL-6 (14). Microbiota metabolites also affect immune cell activity. For example, the SCFAs produced by beneficial symbiotic bacteria can increase the antitumor response of CD8^+^ T cells (96). SCFAs can reduce the down-regulation of macrophage pro-inflammatory mediators such as IL-6, thereby inhibiting tumor growth (58). Unfortunately, in PC patients, the beneficial symbiotic bacteria that produce SCFAs are reduced, thereby subduing the antitumor response of CD8^+^ T cells and indirectly promoting the tumor-promoting effect of macrophages. In addition, not all SCFAs are beneficial. For example, butyrate can increase regulatory T cells (Treg) production (97). Treg cells are considered one of the most effective antitumor immunosuppressants, capable of reducing the activity of CD4^+^, CD8^+^ and NK cells, and are associated with poor prognosis in PC (98). In short, the microbiota might regulate the proportion of immune cell components in the TME (**Figure 5**), which indirectly affects the tumor immune response, thereby changing the malignant phenotype of the tumor.

### The Pro-Inflammatory and Anti-Inflammatory Imbalance Caused by Microbiota

Microbiota metabolites mediate the communication between the symbiotic microbiota and the immune system, affecting the balance between pro-inflammatory and anti-inflammatory mechanisms (99). As mentioned above, in chronic pancreatitis tissues, T cells and macrophages are the main immune infiltrating cells (62, 63). Macrophages are another type of immune cells that can differentiate into two phenotypes, M1 and M2. They are usually responsible for removing debris from injury or infection sites and present antigens to the host’s immune cells (B and T cells), triggering adaptive immunity. It has been shown that in early tumors, TAMs exhibit more pro-inflammatory M1 phenotypes that promote antitumor activity, and as the disease progresses, they exhibit more M2 phenotypes, that is, anti-inflammatory and contribute to tumor immunoediting, facilitating tumor growth and invasion (99). In the mouse PC model, Zhu et al. found that PC tissue macrophages were significantly increased, which promoted the development of high-grade aggressive tumors and the proliferation of PC cells and the pro-fibrotic phenotype (100). M1 secretes pro-inflammatory factors IL-1β, IL-6, TNFα (99). However, these pro-inflammatory factors with antitumor effect can also become a tumor-promoting factor in a certain environment. For example, in a mouse model of PC liver metastasis, IL-6 derived from activated macrophages activates the oncogenic transcription factor STAT3, which directly inhibits the miR-124 gene through its conserved STAT3 binding site in its promoter; targeting Notch ligand Jagged 1 to regulate Notch signaling in cancer cells, thereby promoting mesenchymal transition and invasion (101). The pro-inflammatory factor TNF-α relies on the NF-κB pathway to regulate the expression of GLI1 to induce EMT phenotype, malignant behaviors and drug resistance (102). However, the high levels of innate cytokines in chronic inflammation may induce angiogenesis, cancer cell migration, and EMT by driving sustained NF-κB activation and mitogen-activated protein kinase (MAPK) activity (89, 103, 104). For example, M2 macrophages express high levels of anti-inflammatory cytokines (such as IL-10, TGF) to promote cancer cell metastasis (105, 106). *In vivo* and *in vitro* experiments revealed that M2 macrophages secrete CCL18 which specifically binds to PITPNM3 at the cellular membrane and subsequently upregulates VCAM-1 expression in PC cells by activating NF-kB signal transduction, promoting the Warburg effect, proliferation, migration and metastasis of PC cells. In that study, the authors found that VCAM-1-derived lactic acid could promote the M2-like polarization of macrophages in a dose-dependent manner, indicating a mutual feedback loop between CCL18-positive macrophages and PC cells (107). Wnt pathway activation is also related to macrophage activation and is a driver of PC development (108). In addition, the polarization of M2 macrophages could lead to the increased metastatic potential of PC cells (109). In the TME, CD4^+^ T cells recognize the exogenous antigen peptides presented by MHCII molecules. After activation, they will mainly differentiate into Th and mature into Th1, Th2, Treg or Th17 lineage, classic Th1 cytokine-interference IFNγ plays an antitumor effect in TME, and Th2, Treg, Th17, cytokines-IL-4, IL-5, IL-10 and IL-17a mediate tumor-promoting effects (87). For instance, Th17 CD4^+^ cells release the pro-inflammatory factor IL-17, which causes adenocarcinoma cells to increase the activation of NF-κB and mitogen-activated protein kinase signals and increase the expression of DCLK1 and ALDH1A1 (markers of embryonic stem cells). In human pancreatic tumor tissues, patients with high levels of DCLK1 have a shorter median survival time (88). In PC patients, tissues infiltrated by CD4^+^ T cells show a shorter survival time (90). CD8^+^ T cells recognize endogenous antigen peptides presented by MHC class I molecules and differentiate into cytotoxic T lymphocytes (CTLs) after activation. In the pancreatic tissue of longer-lived patients, CD8^+^ T cells have higher infiltration, which is beneficial to the clinical outcomes of patients (90). The microbiota may induce the differentiation of tumor-promoting immune cells and hinder the differentiation of antitumor immune cells (**Figure 5**), thereby causing an imbalance between pro-inflammatory and anti-inflammatory factors (14). In addition, the complement system plays an important role in the proliferation, migration, invasion and EMT of various tumors (93). For example, the presence of fungi in PC tissues promotes tumor growth through the mannose-binding lectin (MBL)-C3 complement cascade pathway (15).

## Implications of the Microbiota in the Treatment of PC

### Microbiota and Chemotherapy

At present, although gemcitabine is still the standard first-line choice for advanced PC, its benefits on the survival of PC patients remain non-ideal (110). One of the reasons may be that the microbiota damages the antitumor properties of gemcitabine. Geller et al. proved that most of the microorganisms associated with pancreatic tumors are *Gammaproteobacteria*, including *Enterobacter* and *Pseudomonas*, which can produce cytidine deaminase (CDD) and promote the metabolism of gemcitabine into its inactive form, 2’,2’-difluorodeoxyuridine, leading to the degradation and resistance of gemcitabine (12). The combination of gemcitabine and antibiotics is more effective than gemcitabine alone. Thus a possible mechanism of this action might be because the bacteria in the tumor reduced the metabolism of gemcitabine (111). In addition, the pyrimidine nucleoside phosphorylase (PyNP) produced by mycoplasma can remove the natural pyrimidine nucleosides uridine, 2’-deoxyuridine and thymidine, which indirectly affects the therapeutic effect of chemotherapy drugs (112). The above experimental results illustrate that the microbiota, including bacteria and mycoplasma, may be the main culprit for the poor efficacy of gemcitabine, a chemotherapy drug for PC. However, the culprits discovered are just the tip of the iceberg.

### Probiotics Combined With Chemotherapy

Probiotics are live microorganisms, which confer a health benefit on the host and most frequently belong to the lactic acid bacteria categories *Lactobacillus spp*. and *Bifidobacterium spp* (113). Using a mouse model of PC xenotransplantation, Panebianco et al. found that probiotics combined with chemotherapy can significantly increase the DNA damage of PC cells, effectively inhibit the cell cycle, and induce cell apoptosis, and effectively inhibit the EMT of PC cells, and better preserve the overall structure of the intestinal mucosa, and increase the species richness of the intestinal microbiota, which is mainly manifested in the bacteria that produce butyrate and other beneficial SCFAs, such as *Eubacteriaceae*, *Ruthenibacterium*, *Faecalicatena*, *Pseudobutyrivibio* and *Roseburia*. In addition, probiotics restore the number of platelets affected by gemcitabine (114). Combining gemcitabine and probiotics can cause a reduction in the formation of PanIN and the expression of vimentin and Ki-67. Mice treated with gemcitabine combined with probiotics have lower aspartate aminotransferase (AST) and alanine aminotransferase (ALT) levels. These findings indicate that probiotics can make standard chemotherapy more effective and help improve patients’ tolerance to chemotherapy (115). In addition, probiotic administration reduced the histological expression of Smad3 and phosphorylated Smad3 in KC mice treated with Porphyromonas gingivalis. It may have beneficial effects by reducing cancer cell proliferation and viability, inhibiting PanIN progression, EMT and cancer cell metastasis (116). In a xenograft model, the probiotic bacteria *Lactobacillus casei*-derived iron pigment could suppress the progression of cancer cells and induce apoptosis of PC cells by activating p53 and hindering the cell cycle. Its antitumor efficacy was even better compared to a combination of 5-FU and cisplatin in refractory and resistant PC (117). However, the application of chemotherapeutic drugs can also change the microbiota and its metabolites in PC patients. An overall increase in inflammation-related bacteria was observed with gemcitabine. Besides, activation of the NF-kB classic pathway was found in the cancer tissues of mice treated with gemcitabine (118). Therefore, the microbiota could affect the therapeutic efficacy of chemotherapeutics. Importantly, the elimination of pathogenic bacteria and probiotic application could increase the efficacy of chemotherapeutics and improve patients’ tolerance to chemotherapy. Conversely, chemotherapeutic drugs could cause a change in the composition of the microbiota, leading to a vicious circle and ultimately accelerating tumor progression. However, the conclusion that probiotics contribute to the treatment of PC patients needs more data to support.

Prebiotics, a substrate selectively utilized by host microorganisms, confer health benefits, mainly include indigestible fructo-oligosaccharides (FOS) and galactans (GOS), which are preferentially metabolized by *Bifidobacteria* to convert them into SCFAs, namely propionate, butyrate and acetate, and they are essential for intestinal health (119, 120). Unfortunately, reports on its connection with PDAC have not been published publicly, but it can be used as a future research direction.

### Microbiota and Immunotherapy

Immune cells are essential in the PC microenvironment. On the one hand, immune cells can recognize and kill tumor cells; on the other hand, immune cells and their related inflammatory factors can promote tumor occurrence and progression (121). Antitumor immune cells and tumor-promoting immune cells in the microenvironment of PC are reportedly regulated by the microbiota. In terms of mechanism, the microbiota can act as an antigen and activate the immune system. More importantly, in the absence of intestinal flora, the immune system cannot be activated (122, 123). For example, MDSC has antigen-non-specific and antigen-specific immunosuppressive effects. Once it enters the TME, it can cause oxidative stress on surrounding immune cells and inhibit T cell proliferation, and the MDSC level of patients was found to be higher than that of healthy people (124). Antibiotic ablation could reduce MDSC in mouse PC tissue (14), which indirectly reflects the influence of the microbiota on MDSC infiltration of PC. In the TME, CD4^+^ T cells differentiate into Th upon activation and mature into the Th1, Th2, Treg or Th17 lineage. The classic Th1 cytokine-IFNγ plays an antitumor effect while Th2/Treg cytokines-IL4, IL5, IL10, IL17, etc., have tumor-promoting effects (87). After activation, CD8^+^ T cells differentiate into CTLs, which have antitumor effects (89). Interestingly, PC patients showing a higher degree of CD8^+^ T infiltration possessed a longer survival time (90). The microbiota in the TME induces the production of tumor-promoting immune cells Th2, Treg or Th17 while inhibiting antitumor immune cells. Treg is considered one of the most effective antitumor immunosuppressants, being able to effectively reduce the activity of CD4^+^, CD8^+^ and NK cells. Several experimental results have shown that the immunomodulatory molecule polysaccharide A (PSA) of *Bacteroides fragilis* can mediate the transformation of CD4^+^ T cells into Foxp3(+) Treg cells and produce IL-10 during symbiotic colonization (125). The ablation of the microbial population could improve this outcome and enhance the infiltration of antitumor immune cells (14, 15, 87).

Immune checkpoint molecules (immune checkpoint) are inhibitory regulatory molecules in the immune system, which are essential for maintaining self-tolerance, preventing autoimmune responses, and minimizing tissue damage by controlling the duration and intensity of immune responses. Immune checkpoint molecules expressed on immune cells can inhibit the function of immune cells and prevent the body from producing effective antitumor immune responses, leading to immune evasion. Currently, cytotoxic T lymphocyte-associated antigen 4 (CTLA4) and programmed cell death protein 1 (PD-1) are being actively researched. Immune checkpoint blocking (ICB) immunotherapy targets intrinsic immune downregulation factors, such as CTLA4, PD-1 and PD-L1, leading to a lasting clinical response and has recently become a source of promising new cancer treatments (126, 127). Recently, several studies have shown that the gut microbiota can enhance the antitumor efficacy of PD-1 and CTLA4 blocking therapies (128). In addition, experimental results show that immune checkpoint inhibition (ICI) therapy and adoptive cell therapy using tumor-specific CD8^+^ CTL are affected by the gut microbiota composition (129, 130). Intestinal bacteria capable of producing SCFA, including eubacteria, lactobacilli and streptococci, are positively correlated with the anti-PD-1/PD-L1 response of different gastrointestinal cancer types (131). The efficacy of checkpoint blocking immunotherapy has been proven to depend on the presence of unique beneficial bacteria in the patient’s intestines. Oral bifidobacteria can improve the tumor control of PD-L1 specific antibody therapy. The effect is that oral bifidobacteria alone improves tumor control to the same extent as PD-L1 specific antibody therapy (checkpoint blockade), and the combination therapy almost eliminates tumor growth. This effect is mediated by enhanced dendritic cell function leading to enhanced CD8^+^ T cell priming and accumulation in the TME (132). Mager et al. isolated three kinds of bacteria, namely *Bifidobacterium pseudolongum*, *Lactobacillus johnsonii* and *Olsenia*, which significantly enhanced the efficacy of immune checkpoint inhibitors in mouse models of cancer (133). Although ICB immunotherapy stimulates T cell activation and effective antitumor immune response, it may also cause severe inflammatory side effects in some patients, termed immune-related adverse reactions (irAE), similar to autoimmune diseases. IrAEs are common and can occur in up to 90% of patients treated with anti-CTLA4 antibodies (134) and 70% of patients treated with PD-1/PD-L1 antibodies (135). Although any organ system can be affected, irAE most commonly involves the gastrointestinal tract, endocrine glands, skin, and liver (135). One of the common toxicities is immune checkpoint block-associated colitis (136).

### Microbiota and Radiotherapy

Radiotherapy is one of the important methods for the treatment of PC, especially suitable for patients with advanced PC (137). Radiotherapy may enhance the release and absorption of tumor-associated antigens, thereby promoting the initiation of anti-tumor T cells, and enhancing entry into the tumor due to the impact on the tumor vascular system and the chemokine environment (138). Interestingly, as mentioned above, the microbiota plays an important role in the immune microenvironment of PC. It can be assumed that the intestinal flora also plays a role in the immune stimulating effects of radiotherapy. However, radiotherapy is a double-edged sword, while killing tumor cells, it also affects the healthy tissues of the body, mainly manifested in the damage to the bone marrow and digestive tract mucosa (139). One of the reasons for this result may be that the composition of the body’s digestive tract microbiota has been changed after radiotherapy (140). The application of probiotics improves the tolerance of PC patients to radiotherapy. Experimental results prove that preparations containing probiotics such as *Lactobacillus* and *Bifidobacterium* have a protective effect on radiotherapy-induced intestinal toxicity and can significantly reduce the incidence of severe diarrhea (141, 142). To date, little is known about the response caused by microbiota to radiotherapy in PC and reliable data is needed to prove that some microbiota is beneficial in radiotherapy.

### Antibiotic Application

The combination of antibiotics and chemotherapeutics indeed enhances the antitumor efficacy of chemotherapeutics and helps to improve the tolerance of patients to chemotherapy. For example, Weniger et al. found that the progression-free survival (PFS) of some PC patients was not improved after adjuvant gemcitabine treatment after surgery. The intraoperative bile culture of these patients found that *Klebsiella pneumoniae* was positive, and the survival time was significantly improved after quinolone treatment (143). Similarly, a previous study shown that fungal ablation could enhance the effect of gemcitabine-based chemotherapy (15). However, the use of antibiotics is not all beneficial. For instance, long-term use of tetracycline increases the risk of prostate cancer and breast cancer (144). The use of penicillin is closely related to the occurrence of PC (145). This may be because the use of antibiotics changes the composition and proportion of the microbial population, rendering some opportunistic pathogenic bacteria more resistant, leading to more pathogenic strains, thereby promoting tumor development. Unfortunately, previous large-scale clinical studies did not dynamically track changes in the microbiota during antibiotic use. In addition, the metabolic changes caused by antibiotics may also play a critical role in promoting tumors (144).

### Fecal Microbiome Transplantation

As mentioned earlier, the microbiota (including those in the intestine) can migrate to the pancreatic tissue through a variety of ways, which provides a way for the fecal microbiome transplantation (FMT) to assist the treatment of PC. Riquelme et al. demonstrated that the use of FMT in an antibiotic-treated mouse model causes the gut microbiota to colonize pancreatic tumors and change the overall bacterial composition of the tumor (48). Compared with STS, the microbiota, FMT from the LTS cohort, induced anti-tumor response and immune system activation in mouse PC tissue, characterized by infiltration with cytotoxic CD8^+^/killer T cells (48). However, the potential risks of FMT, including the possible transmission of pathogens to recipients, should not be ignored. Patients are stratified to screen specific microbiota, especially microbiota that can improve the efficacy of anti-cancer drugs, to choose more effective treatments and reduce treatment complications.

## Conclusions

In less than a decade, the mechanism of action of the microbiota, the second-largest gene pool of the human body, in PC has been increasingly studied. In addition to the interaction between PC and microbiota, current research on the tumor-promoting and antitumor effects of microbiota and its metabolites in PC has also focused on the “micro-immune system” changes in the TME. However, due to the large variety and the huge number of microbiota and its symbiosis in the human body, it has received many aspects, such as age, gender, immune ability, diet, climate, and regional influences, making the use of microbiota for the precise treatment of PC fraught with challenges. There is still a long way to go before the development and implementation of an efficacious and robust microbiota-related precision treatment. This review offers much-needed insight into the various mechanism and current therapeutic advances in microbiota-associated PC, aiming to provide the impetus for further in-depth studies.

## Author Contributions

ZC, SZ, and WZ conceived the review. ZC, SZ, SD, and HX undertook the initial research. ZC, SZ, SD, and HX were involved in writing. WZ reviewed the manuscript. All authors contributed to the article and approved the submitted version.

## Funding

This article was supported by The First Hospital of Lanzhou University Intra-Hospital Fund Youth Fund, ldyyn2020-76.

## Conflict of Interest

The authors declare that the research was conducted in the absence of any commercial or financial relationships that could be construed as a potential conflict of interest.

## Publisher’s Note

All claims expressed in this article are solely those of the authors and do not necessarily represent those of their affiliated organizations, or those of the publisher, the editors and the reviewers. Any product that may be evaluated in this article, or claim that may be made by its manufacturer, is not guaranteed or endorsed by the publisher.
